# Porcine Monocyte DNA Traps Formed during Infection with Pathogenic *Clostridioides difficile* Strains

**DOI:** 10.3390/pathogens13030228

**Published:** 2024-03-05

**Authors:** Jade Lawrence, Paul Barrow, Neil Foster

**Affiliations:** 1 School of Veterinary Medicine and Science, University of Nottingham, Nottingham LE12 5RD, UK; jade_drew_lawrence@hotmail.co.uk; 2 School of Veterinary Medicine, University of Surrey, Daphne Jackson Road, Guildford GU2 7AL, UK; paul.barrow@surrey.ac.uk; 3 Department of Veterinary and Animal Science, SRUC Aberdeen, Craibstone Campus, Aberdeen AB21 9YA, UK

**Keywords:** *Clostridioides* (*Clostridium*) *difficile*, monocyte, DNA traps, etosis, pigs

## Abstract

*Clostridioides* (*Clostridium*) *difficile* is an enteric pathogen of several mammalian species including man, frequently involving nosocomial resurgence, following oral administration of broad-spectrum antibiotics, but also with human-to-human infection occurring, and neonatal pigs with zoonotic transmission. To date, the immune response to *C*. *difficile* has mostly focused on neutrophils and cytokine/chemokines, particularly in human infection. The neonatal pig is now recognized as a valuable model for human infection. We show that porcine monocytes respond to *C*. *difficile* differently compared with many other bacterial infections. Infection of porcine monocytes with human *C*. *difficile* strains CD630 (Ribotype 078) or R20291 (Ribotype 027) for 3 or 24 h post-infection (pi) resulted in a lack of oxidative burst or nitrite ion production when compared to uninfected controls (*p* > 0.05). The survival dynamics of both CD630 and R20291 in monocytes were similar with intracellular bacterial numbers being similar at 3 h pi and 24 h pi (*p* > 0.05). However, we show that porcine monocytes entrap *C*. *difficile* via extracellular DNA traps. This process began as early as 3 h pi, and at 24 h pi the nuclei appeared to be depleted of DNA, although extracellular DNA was associated with the cell membrane. Our preliminary study also suggests that entrapment of *C*. *difficile* by extracellular DNA may occur via a process of monocyte etosis.

## 1. Introduction

*Clostridioides difficile* is a Gram-positive, spore-forming, anaerobic enteropathogen of humans, pigs, and other animals [1,2,3] although it can also be isolated from clinically normal individuals.

In man, it has been typically associated with elderly and immune-compromised patients in a hospital or health care-home setting, and frequently within a few days of receiving orally administered broad-spectrum antibiotics, sometimes in preparation for surgery [4]. Suppression of the normal protective microbiome (dysbacteriosis) facilitated multiplication of *C. difficile*, and transmission between patients also occurred before more stringent hygiene measures were introduced. Most cases now occur outside of the health care environment and more frequently in younger people. *C. difficile* in pigs is now recognized as an important source of human infection [5] and could spread antimicrobial resistance into the human food chain.

Pathology occurs in the colon with severity varying between mild diarrhea to severe pseudo-membranous colitis with mortality. Pathology results from the production of Rho-glucosylating toxins A and B (TcdA and TcdB), which induce a significant inflammatory immune response and subsequent cellular pathology.

In pigs, *C. difficile* is a common cause of diarrhea in animals less than one week old showing in severely affected animals, at necropsy, inflammation of the large intestine, a pseudomembrane, and colonic mucosal hemorrhage, which can extend from the ileo–cecal/colon junction to the rectum together with the characteristic volcano lesions. The lesions are identical to those seen in human infection. It is therefore hardly surprising that the pig model has been advocated as a good and representative model of human disease [6] in addition to the more frequent earlier use of the hamster and murine models [7,8].

Much of the focus on the immune response to *C*. *difficile* has centered on the role of neutrophils and inflammatory mediators such as cytokines, [9] particularly in the development of classical volcano lesions in colonic tissue [10]. However, monocytes play a key role during intestinal inflammation. In a dextran sulphate-induced murine model of colitis, F4/80^+^CD11b^+^CCR2^+^Ly6C^high^ inflammatory monocytes were recruited to the colon and F4/80^+^CD11b^+^CCR2^+^Ly6C^high^ colonic monocyte/macrophages correlated with colonic eosinophilic inflammation [11]. In experimental murine *C*. *difficile* infections, innate cells including monocytes are recruited to the large intestine [12,13] in a CCR2-dependent manner [14]. This indicates that monocyte chemoattractant protein 1 (CCL2) and its receptor are essential in this process, although CCR deficiency does not alter the course of infection [14].

Although relatively little is known about the immunological function of monocytes in the colonic epithelium during *C*. *difficile* infection, a study by McDermott et al. [15] reported that, in mice, CCR2 and IL-23 are required for monocyte recruitment, and this increases inducible nitric oxide synthase (INOS) expression in colonic tissues. INOS is a precursor required for reactive nitrogen species (RNS) such as NO_2_^−^, which is a known effector in many different types of bacterial infection. However, toxin A from *C*. *difficile* strain 10,463 has been shown to inhibit reactive oxygen species (ROS) production by TNF-α-stimulated human neutrophils [16], and ROS is another common killing pathway utilized by innate immune cells during bacterial infection. However, nothing has been reported regarding the response of porcine monocytes to human *C*. *difficile* strains.

The aim of this preliminary study was to describe the behavior of porcine monocytes in response to *C*. *difficile* CD630 (Ribotype 078) or R20291 (Ribotype 027) infection, both of which have been shown to be pathogenic in pigs and humans [17].

## 2. Materials and Methods

### 2.1. C. difficile Strains and Culture

Two pathogenic strains of *C. difficile* were used, CD630 and R20291, received from Professor Nigel Minton (School of Molecular Medical Science, The University of Nottingham). CD630 was initially sourced from a patient in Switzerland suffering from severe pseudomembranous colitis in 1982. R20291 (a hypervirulent RT078 strain isolated frequently from man and animals) was sourced from an outbreak in 2006 at Stoke Mandeville hospital, UK [18]. 

Strains were stored as spore preparations at 4 °C, which were cultured on *C. difficile* Agar Base (Oxoid Ltd., Hampshire, UK), supplemented with cholic acid sodium salt (Acros Organics, Antwerp, Belgium) 0.1% (*w*/*v*) to aid germination, which was autoclaved and cooled to ~40 °C before 7% (*v*/*v*) pre-warmed defibrinated horse blood was added (Oxoid). The strains were incubated anaerobically at 37.4 °C for 2–3 days in a Bactron™ Environmental Chamber (Bactron II-2, Sheldon Manufacturing Inc., Cornelius, OR, USA). Both strains were passaged twice-weekly for preparation of liquid cultures.

Liquid cultures for experimental use were prepared using anaerobe basal broth (Oxoid), which was taken immediately from the autoclave, with pressure released manually, to the anaerobic chamber, to prevent oxygen infusing back into the liquid. Broths were held in the port for 5 days, with loose lids, before inoculation from the passaged strains. Broths were incubated at 37 °C for 2 days prior to use. 

Bacterial cell/spore concentration was determined by enumeration of colony-forming units (CFU/mL) [19]. Serial dilutions were plated out on unsupplemented *C. difficile* agar, cholic acid, and defibrinated horse blood (CCH) plates in the environmental chamber. These were incubated for 24 h and then counted. Work on the different strains was performed in different days to reduce any risks of cross contamination. No other strains were in use in the laboratory.

### 2.2. Porcine Monocyte Isolation and Culture

Whole blood with lithium heparin was purchased from Matrix Biologicals Ltd. (Hull, UK) obtained from 8 clinically healthy, 5–6 months old large white female pigs at slaughter. Standard density-gradient centrifugation of whole blood from each sample was performed using Histopaque (Sigma, Dorset, UK) to obtain buffy coats. The resulting pellet was resuspended in PBS for cell enumeration, using a hemocytometer and Trypan Blue Solution 0.4% (Sigma, Solent, UK) to analyze cell viability. This showed that >99% cells were viable in all the buffy coat samples analyzed. A total of 1 × 10^6^ buffy coat cells were seeded into 500 µL of RPMI 1640 (Gibco, Thermo Fisher Scientific, Winsford, UK), L-Glutamine (1% *w*/*v*, Gibco) combined with fetal calf serum (5% *v*/*v*), and penicillin and streptomycin, per well of a 24-well cell culture dish (Nunc, Naperville, IL, USA). Cells were cultured for 2 h at 37 °C and 5% CO_2_, after which supernatants were removed and attached cells (monocytes) were then detached gently and cultured in fresh RPMI media at 1 × 10^5^ monocytes per 500 μL for 24 h prior to use.

### 2.3. Fluorescence-Activated Cell Sorting (FACS)

FACS was performed in order to determine CD14 expression (monocytes) within the buffy coat. Buffy coats were washed in PBS, centrifuged for 5 min, and resuspended in 100 µL PBS to give 1 × 10^6^ cells. 10 µL (10 µg/mL) of mouse anti-porcine CD14 antibody conjugated to fluorescein isothiocyanate (FITC) (AbD Serotec, Oxford, UK) was added to the sample and incubated in a dark room at 4 °C for 30 min. Mouse IgG2β FITC (AbDSerotec, Kidlington, UK) was used as an isotype control. After 30 min, samples were washed in 5 mL PBS and centrifuged for 5 min prior to being resuspended in 500 µL of PBS for CD14 expression analysis by FACS (FACS Canto II™ Flow Cytometer, Becton Dickinson and Company, Winnipeg, MB, USA). 

### 2.4. Monocyte Infection Studies

A total of 1 × 10^6^ cells of each *C. difficile* strain was added to monocyte cultures to achieve a multiplicity of infection (MOI) of 10 and incubated for 2 h; negative control cells were incubated with media only. After 2 h, the wells were washed 3 times in PBS warmed at 37 °C, and the monolayer was inspected using an upright microscope to assess cell integrity. Cells were then incubated with vancomycin for 60 min to kill extracellular bacteria as carried out previously with *Salmonella* and gentamicin [20]. Cells were incubated for either 3 or 24 h post-infection (pi) prior to washing a further 3 x in PBS before all the liquid was removed from the cells. The plates were then transferred to the anaerobic environmental cabinet and lysed with 0.5% Triton-X-100 for 30 min. Serial dilutions were carried out plating on CCH plates. The test was performed in triplicate for each of the 8 pigs. 

### 2.5. Measurement of ROS in Monocytes

A standard nitroblue tetrazolium (NBT) reduction assay [21] was used to measure ROS activity in porcine monocytes at 3 and 24 h pi. Briefly, infected and control monocytes were incubated for 45 min with 50 μL NBT (Sigma, Solent, UK) (10 mg/mL NBT in PBS) at 37 °C in 5% (*vol*/*vol*) CO_2_. The cells were then washed in PBS and incubated in 100 μL 1M hydrochloric acid for 10 min to stop the reaction. The cells were then washed in PBS, and 150 μL dimethyl sulfoxide (Sigma, Solent, UK) was added and mixed thoroughly prior to addition of 10 μL 5 M sodium hydroxide to develop the color. The optical density of the reaction mixture was determined at 620 nm with a plate reader (Anthos Labtech Instruments, Hamburg, Germany). As a positive control, monocytes were incubated with 1 μg/mL zymosan (Sigma, Poole, UK) for the same times.

### 2.6. Measurement of RNS in Monocytes

At 3 h and 24 h pi, supernatants from infected and uninfected monocytes were removed and stored at −80 °C prior to use. Greiss reagent (Promega, Madison, WI, USA) was used to detect nitrite ions in the supernatants according to the manufacturer’s instructions measuring absorbance at 540 nm. As a positive control, monocytes were incubated with 10 μg/mL phorbol myristate acetate (PMA) (Sigma, Poole, UK) with the manufacturer’s guidelines, and absorbance was measured at an optical density of 540 nm. 

### 2.7. Fluorescence Microscopy

Autoclaved, circular, glass coverslips were placed on the bottom of 24-well plates. Monocytes were seeded onto the glass as described in Section 2.2, and *C. difficile* infection was carried out as described in Section 2.3. At 3 h and 24 h pi the coverslips were removed from the wells and fixed in acetone for 15 min. The coverslips were then washed 3 times in PBS. Phalloidin (10 mg/mL) was used to stain actin within the cells for 15 min. The coverslips were placed on microscope slides and mounted with ProLong Gold anti-fade reagent with DAPI (Invitrogen, Paisley, UK) before covering with a square coverslip. All slides were examined using a TCS SP2 confocal microscope (Leica Microsystems, Heidelberg, Germany).

The *C*. *difficile* strains were also examined by fluorescence microscopy. A loop of each *C. difficile* broth culture was diluted 1:10 and smeared onto a microscope slide. The preparations were stained with phalloidin as detailed above and mounted on a slide using DPX-mountant (Sigma, Poole, UK) with a cover slip prior to examination using a TCS SP2 confocal microscope (Leica, Wetzlar, Germany). This approach has been used regularly by this group [22,23].

### 2.8. Statistical Analysis

Statistical analysis was performed by Student’s *t*-test using GraphPad Prism version 5.00 for windows (GraphPad Software, San Diego, CA, USA). Statistical significance was assessed at the 5% confidence limit (*p* < 0.05)

## 3. Results

### 3.1. Phenotype and Morphology of Uninfected (Steady-State) Porcine Monocytes

**Figure 1:**
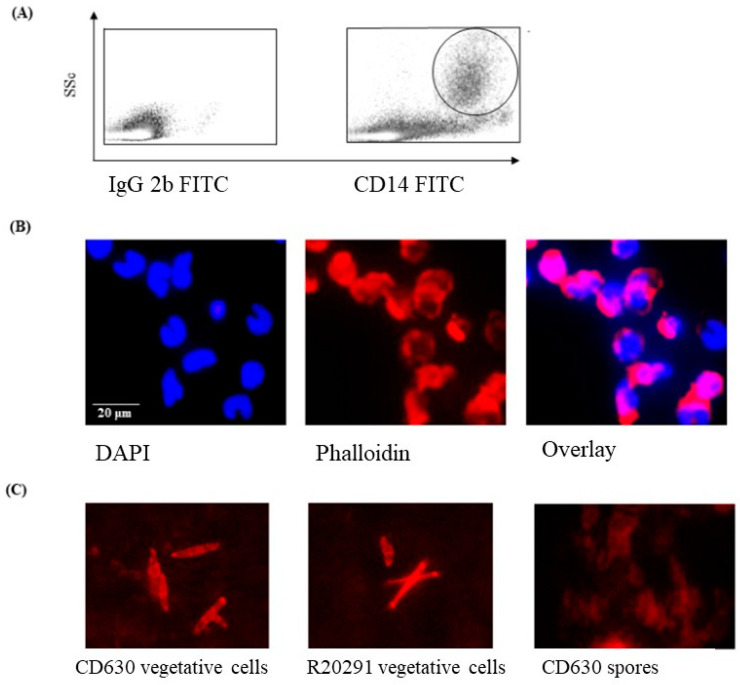
Morphological appearance of porcine monocytes and *C. difficile* vegetative cells and spores. (**A**) FACS analysis of uninfected porcine monocytes showing a SSc ^high^/CD14^high^ phenotype (large cells expressing CD14) typical of monocytes. (**B**) DAPI staining showing the typical densely packed nuclear DNA in uninfected macrophages with a “C-shaped” morphology with Phallodin marking the extent of the cell outlines. (**C**) Phalloidin staining of cultures and spores of *C*. *difficile* strain CD630 and R20291 showing oval to elongated bacilli. Bacteria spores are notoriously difficult to stain, and this was expected to be the case with *C. difficile* spores.

Initial FACS experiments were performed to determine the morphology of the monocytes prior to infection with *C. difficile* strains CD630 or R20291. The cells were large (SSc high) and expressed CD14 (Figure 1A) and CD172a (not shown). The high SSc/CD14 staining indicated that the cells cultured were monocytes. The nuclei of the cells were typically C-shaped with densely packed DNA, shown by DAPI staining (Figure 1B). 

### 3.2. Staining of C. difficile Vegetative Cells

Phalloidin staining of bacterial cells showed a clearly discernable but mixed morphology consisting primarily of medium to long bacilli, which was observed consistently in preparations of both *C. difficile* CD630 and R20291 strains (Figure 1C). In contrast, phalloidin staining of the storage spore preparation was not at all clear, which is likely consequence of the spores not having taken up the stain at all. 

### 3.3. Survival Dynamics of C. difficile in Porcine Monocytes

**Figure 2:**
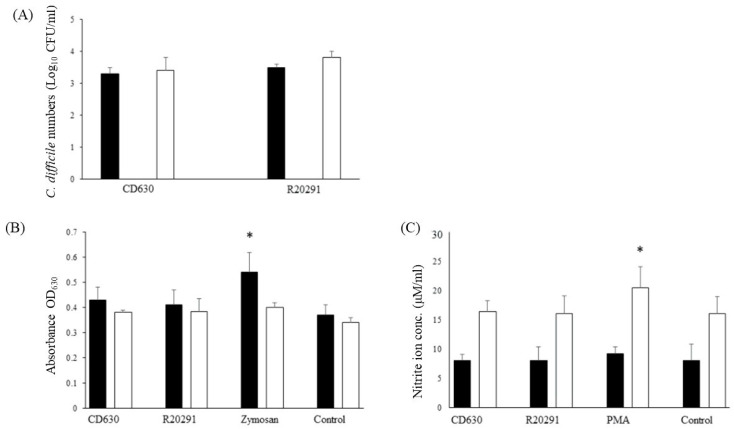
Survival of *C*. *difficile* CD630 and R20291 in porcine monocytes and production of ROS and RNS. (**A**) The numbers (CFU/mL lysate) of *C*. *difficile* CD630 and R20291 recovered after 3 h and 24 h infection of porcine macrophages. (**B**,**C**) Production of reactive oxygen species (**B**) detected by NBT or reactive nitrogen species (**C**) detected by Griess reagent. Black bars = 3 h pi; White bars = 24 h pi. Positive controls were zymosan (ROS) or PMA (RNS). Error bars show the standard deviation from the mean. * = significant difference (*p* < 0.05) from uninfected/unstimulated control monocytes. Data shown were calculated from mean values obtained from 8 pigs, each performed in triplicate.

Survival of both strains in porcine monocytes was good with very little change in the bacterial numbers recovered between 3 h and 24 h albeit with a very small non-significant increase at 24 h (Figure 2A). 

### 3.4. Reactive Oxygen and Nitrogen Species Produced by Porcine Monocytes in Response to C. difficile Infection

Oxidative burst tends to be more acute than the production of nitric oxide. The product of the NBT reaction indicated that levels of reactive oxygen species produced were similar to those of the uninfected monocytes whereas significantly more was produced by the positive zymosan control (Figure 2B). 

Similar results were found for reactive nitrogen species with values increasing at 24 h, but no difference was found between the levels produced by the two strains compared with the uninfected negative control and the significantly greater amount produced by the PMA positive control (Figure 2C).

### 3.5. Porcine Monocyte form DNA Traps to Immobilize C. difficile

**Figure 3:**
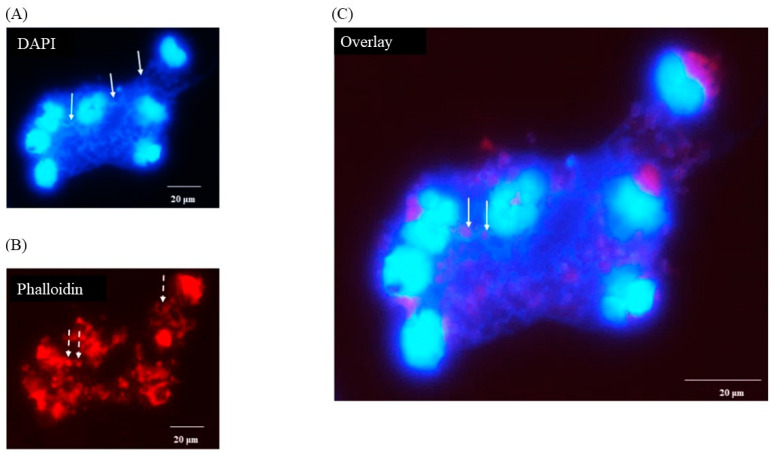
Production of porcine extracellular monocyte DNA in response to *C*. *difficile* infection. DAPI (**A**) and phalloidin (**B**) staining at 3 h pi. Network of DNA ((**A**) solid arrows) inter-connecting infected cells with diffuse outlines ((**B**) broken arrows) clearly visible. Individual bacterial cells possibly visible in overlay ((**C**) solid arrows). Images shown are of *C*. *difficile* CD630 infection and are representative of images taken from monocytes isolated from 3 individual pigs. Similar images observed for strain R20291. Scale bars (20 μm) are shown.

DAPI staining of macrophages 3 h after infection with *C. difficile* CD630 showed a mass of DNA surrounding and connecting groups of monocytes (Figure 3A, solid arrows). The outline of the nuclei of the cells were less distinct than observed in uninfected cells and the cell morphology was also less clear (Figure 3B, broken arrows) when compared with Figure 1B. It was possible in the overlay to observe structures that may have been individual bacterial cells (Figure 3C, solid arrows). None was seen in uninfected cells (data not shown). Similar images were observed for R20291 (data not shown). 

**Figure 4:**
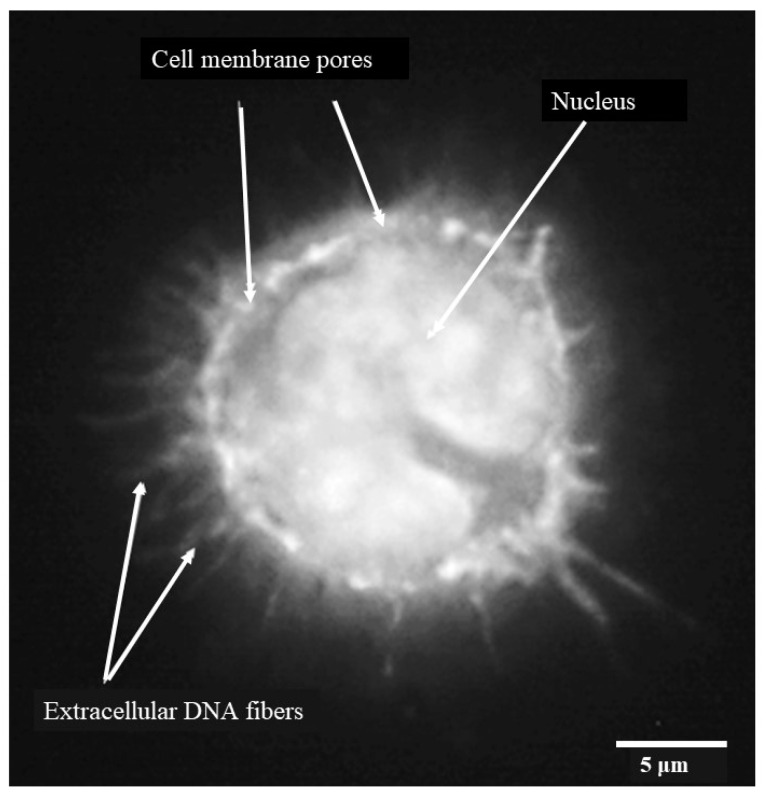
Porcine monocyte etosis at 3 h post-infection with *C*. *difficile* CD630. Higher resolution (grayscale) image of individual infected macrophage stained with DAPI. Fibers of DNA visible protruding through pores in monocyte cell membrane. The classic “C-shaped” morphology was still evident. The scale bar (5 μm) is shown.

When individual monocytes were observed after 3 h, it was possible to discern DNA fibers protruding through pores or discrete areas of lysis in the monocyte cell membrane (Figure 4) indicating early-stage etosis. The outline of the c-shaped nucleus remained clear at this stage.

### 3.6. Following 24 h Culture of Porcine Monocytes with C. difficile, Complete Etosis Occurred but Extracellular DNA Was Still Able to Entrap CD630 or R20291

**Figure 5:**
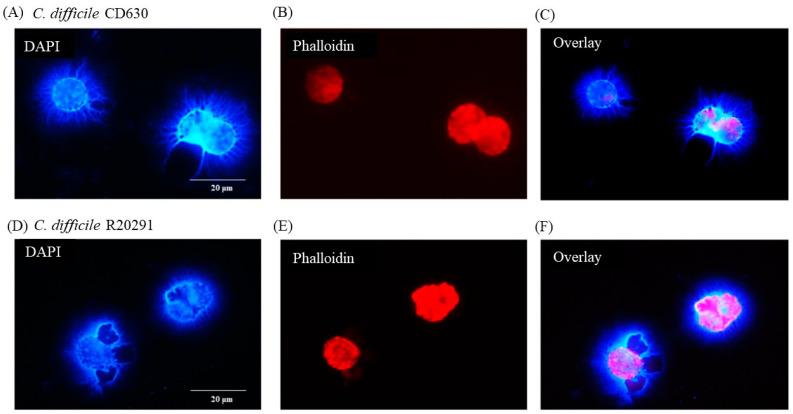
Porcine monocyte etosis at 24 h post-infection with *C*. *difficile*. DAPI (**A**,**D**) and phalloidin (**B**,**E**) staining of porcine macrophages after 24 h infection with *C. difficile* strains CD630 (**C**) and R20291 (**D**–**F**). Images shown are representative of images taken from monocytes isolated from 3 individual pigs. Scale bars (20 μm) are shown.

After 24 h infection, the nuclei were much less clear and, in some cases, not clearly discernible at all. Most of the DNA appeared to be extra-cellular (Figure 5A) with the macrophage cell outline very uneven (Figure 5B,E). The pattern of etosis induced by the two strains appeared to be different with more filamentous release of DNA by CD630 (Figure 5A,C) and more explosive release by R20291 (Figure 5D,F).

**Figure 6:**
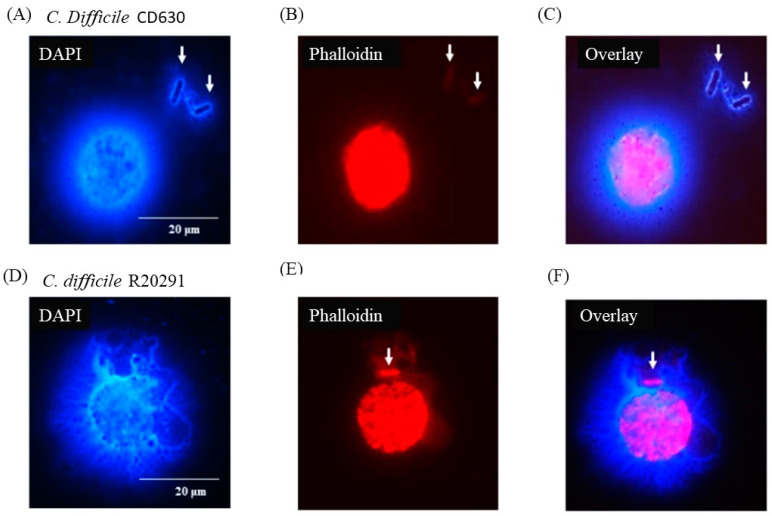
*C*. *difficile* apparently entrapped by DNA released from infected monocytes showing etosis. DAPI (**A**,**D**) and phalloidin (**B**,**E**) staining of porcine macrophages 24 h post-infection with *C*. *difficile* CD630 (**A**–**C**) and R20291 (**D**–**F**). Bacteria entrapped by DNA released from macrophages marked by arrows. Images shown are representative of images taken from monocytes isolated from 3 individual pigs. Scale bars (20 μm) are shown.

Figure 6 shows that individual bacterial cells (solid arrows) not directly in contact with a macrophage appeared nevertheless to be entangled by DNA possibly released by the adjacent monocyte although, in the case of the CD630 (Figure 6C) compared with R20291 (Figure 6F) bacterial cells, this was not completely clear. Uninfected monocytes showed no adverse effects at all at either 3 or 12 h (data not shown).

## 4. Discussion

We report for the first time that porcine monocytes, exposed to the pathogenic *C*. *difficile* strains CD630 and R20291 [17], do not respond by the induction of common (ROS or RNS) innate cell killing pathways but are able to immobilize *C*. *difficile* via DNA traps. Neutrophil extracellular traps (NETS) were first shown to kill Gram-positive and Gram-negative bacteria in a study by Brinkmann et al. [24], and it has remained a contentious issue whether entrapment of bacteria occurs as a consequence of a cell death pathway elicited by bacteria and/or toxins (termed Netosis) or is a neutrophil-driven and evolutionary defense mechanism [25]. Since it is now recognized that other human myeloid immune cells, including macrophages [2] and plasmacytoid dendritic cells [26], can also undergo this process, it is now termed etosis. The proposed significance of etosis for myeloid cells remains unclear, and its potential involvement in innate immunity has also been discussed [27]. In this case, it remains unclear whether bacterial killing might also be taking place or whether the bacteria are simply immobilized facilitating further phagocytosis by neutrophils. 

Our study indicates that the process of monocyte etosis with *C*. *difficile* CD630 or R20291 occurs as early as 3 h post-infection, as indicated by nuclear DNA protruding through pores in the monocyte membrane [28]. At this time point, monocyte DNA surrounded the bacteria and although large numbers were entrapped between groups of monocytes, nuclear DNA remained largely intact and the characteristic “C” shaped morphology was evident. 

At 24 h post-infection, we show that most nuclear DNA was extracellular and although it was still associated with the cell membrane it appeared to be effectively lost from the cell nucleus. This occurred in monocytes that were not in contact with *C*. *difficile* and may thus be a result of toxin release by *C*. *difficile*. This would indicate a progressive etosis of porcine monocytes from around 3 h to 24 h pi when infected with either *C*. *difficile* CD630 or R20291. However, even by 24 h post-infection, extracellular monocyte DNA was still able to entrap *C*. *difficile* and our data, therefore, suggest that the process of etosis is probably pathogen-driven, but a consequence of that is entrapment of some of the bacterial population. This was supported by the fact that there was no detectable decline in bacterial viability during this period. 

There was no detectable ROS or RNS response by these monocytes at either 3 or 24 h pi. The formation and effect of antibacterial free radicals and molecules such as H_2_O_2_ and NO have been studied for many years [29], and more recently their potential therapeutic use as antimicrobials has been explored [30,31]. However, some bacterial species are known to inhibit production of RNS and ROS by innate immune cells, and α-toxin-induced inhibition of ROS has been reported in human neutrophils infected with *C*. *difficile* strain 10,463 [16]. A correlation between human monocyte etosis and inhibition of ROS has also been reported by Webster et al., [32]. In that particular study, it was shown that, when human monocytes were infected with either *Escherichia coli* or *Klebsiella pneumoniae*, etosis occurred with detectable extracellular DNA, but there was no detectable ROS and phagocytosis was inhibited at this point. In contrast, when human monocytes were infected with *Neisseria meningitidis*, etosis did not occur, there was a detectable ROS response, and the monocytes showed prolonged phagocytic activity [33]. Results from our study would therefore suggest that porcine monocytes infected with human *C*. *difficile* strains CD630 and R20291 undergo a similar process to human monocytes infected with *E*. *coli* or *K*. *pneumoniae*. The standard invasion assay used here was developed for pathogenic members of the *Enterobacteriaceae*, particularly *Salmonella* and related organisms, for which an intra-cellular phase involving macrophages is a key stage in the disease process. This is not necessarily the case with *C. difficile* although it is certainly pertinent to the initiation of the immune response to infection. The two-hour exposure stage allows invasion to take place but exposes the monocytes to extra-cellular toxins produced by the initially extra-cellular bacteria. The bacterial cells observed at 24 h entrapped by the DNA are extracellular, and it is impossible to say at this juncture whether these were bacteria that had been released from dying monocytes or whether they were extra-cellular from the outset, surviving the vancomycin treatment used to kill initially extra-cellular bacteria. 

We cannot say what the overall immunological effect of this response has on *C*. *difficile* infection in pigs or humans. However, studies in mice have previously reported that monocyte recruitment is not required for clearance of *C*. *difficile* but that this may increase the inflammatory response and clinical signs of disease [14]. It is, therefore, possible that monocyte etosis, which we report, may be important in this regard. Our study also shows a hitherto unknown fate of porcine monocytes infected with pathogenic *C*. *difficile*. 

In summary, we show that when porcine monocytes are infected with pathogenic *C*. *difficile* strains, they form extracellular DNA traps that immobilize bacteria and that this occurs via a process of etosis, resulting in complete loss of nuclear DNA and monocyte death. This has implications for a better understanding of immune protection against *C. difficile* but also for potential protective mechanisms against colonization of the alimentary tract of humans and pigs by pathogenic *C. difficile* strains. 

Recent studies have indicated that, in the same way that protection of the alimentary tract of newly-hatched chickens against colonization by *Salmonella* strains can be afforded by pre-colonization with a gut flora preparation [34], a similar approach can be used employing cultures of the human patient’s own intestinal microbiome to prevent nosocomial infection with *C. difficile* or by foreign strains [35,36,37].

A variation to this approach in some livestock species involves pre-inoculation with avirulent strains of *Salmonella,* which are able to prevent infection with virulent strains within 12–24 h, as demonstrated in chickens [38] and pigs [39]. In addition to preventing colonization by the avirulent strain, studies in pigs have shown that this approach can induce a rapid form of protection against clinical diarrhea [40] not related to colonization inhibition. This latter protective effect appears to involve an influx of neutrophils and macrophages into the site of colonization in the intestinal mucosa and involves production of antibacterial ROS at the site of the acute infection [41].

Similarly, it has been shown that colonization of hamsters by an avirulent strain of *C. difficile* can prevent establishment by a virulent strain in the intestine when inoculated 24 h later [42] and involving a close association between the avirulent strains and the intestinal mucosa. It would thus be interesting in this context to explore the effect of avirulent strains of *C. difficile* in inducing etosis, which might conceivably contribute to a protective effect via this mechanism in vivo.

## Data Availability

Research data available on request from corresponding author.
